# Using R programming to inform and improve case notification procedures in a local public health unit: Henrique Barrilaro Ruas

**DOI:** 10.1093/eurpub/ckac130.026

**Published:** 2022-10-25

**Authors:** H Barrilaro Ruas, R Capucho, V Melo Ferreira

**Affiliations:** USP, ACES Alto-Tâmega e Barroso, Chaves, Portugal

## Abstract

**Issue/Problem:**

COVID-19 was declared a pandemic in March 2020. Information systems, particularly those related to laboratory testing notification, became extremely important as a mechanism of fast identification of cases. In Portugal, any test performed in a professional setting is of mandatory notification.

For a local public health unit in northern Portugal, much like in other regions worldwide, challenges related to lack of human and material resources were felt over the course of the pandemic.

**Description of the problem:**

In 2021, an intensive surveillance strategy was implemented using up to date notification database analysis through R programming, focusing on simplified data availability for contact tracing team members and accuracy of notifications submitted by laboratories, including verification of individual identifying information.

**Results:**

Some laboratories were identified has having lower data completion rate, which had negative effects on contact tracing timeliness, while others failed to notify tests conducted. Public Health workers warned partners of these failures and worked with them to develop solutions. Interventions included facilitation of access to technologies to notify test results, as well as revision of internal processes to ensure correct patient identification. During the intervention, successful notification rates were increased, and new informal and formal partnerships were developed, leading to faster identification of clusters.

**Lessons:**

Establishing partnerships with stakeholders and developing support systems is beneficial towards epidemiological surveillance efforts. Adequate analysis of notification procedures was an important step towards standardization and correctness of information required for epidemiological surveillance.

**Key messages:**

- Resilient information systems are important for surveillance, especially during a pandemic.
- Partnerships with community stakeholders are essential to timely and adequate epidemiological response.

